# Lack of Platelet-Activating Factor Receptor Attenuates Experimental Food Allergy but Not Its Metabolic Alterations regarding Adipokine Levels

**DOI:** 10.1155/2016/8601359

**Published:** 2016-05-25

**Authors:** Nathália Vieira Batista, Roberta Cristelli Fonseca, Denise Perez, Rafaela Vaz Sousa Pereira, Juliana de Lima Alves, Vanessa Pinho, Ana Maria Caetano Faria, Denise Carmona Cara

**Affiliations:** ^1^Department of Biochemistry and Immunology, Biological Sciences Institute, Federal University of Minas Gerais (UFMG), 31270-901 Belo Horizonte, MG, Brazil; ^2^Departamento de Morfologia, ICB, Universidade Federal de Minas Gerais, Avenida Antônio Carlos 6627, Pampulha, 31270-901 Belo Horizonte, MG, Brazil

## Abstract

Platelet-activating factor (PAF) is known to be an important mediator of anaphylaxis. However, there is a lack of information in the literature about the role of PAF in food allergy. The aim of this work was to elucidate the participation of PAF during food allergy development and the consequent adipose tissue inflammation along with its alterations. Our data demonstrated that, both before oral challenge and after 7 days receiving ovalbumin (OVA) diet, OVA-sensitized mice lacking the PAF receptor (PAFR) showed a decreased level of anti-OVA IgE associated with attenuated allergic markers in comparison to wild type (WT) mice. Moreover, there was less body weight and adipose tissue loss in PAFR-deficient mice. However, some features of inflamed adipose tissue presented by sensitized PAFR-deficient and WT mice after oral challenge were similar, such as a higher rate of rolling leukocytes in this tissue and lower circulating levels of adipokines (resistin and adiponectin) in comparison to nonsensitized mice. Therefore, PAF signaling through PAFR is important for the allergic response to OVA but not for the adipokine alterations caused by this inflammatory process. Our work clarifies some effects of PAF during food allergy along with its role on the metabolic consequences of this inflammatory process.

## 1. Introduction

Food allergy is an immune-mediated response to food that affects approximately 5% of young children and 3% to 4% of adults in westernized countries [1]. It can be divided into non-immunoglobulin E- (IgE-) and IgE-mediated reactions, in which this last type is the one responsible for the majority of food allergic reactions. In IgE-mediated food allergy, also known as type I food allergy, the allergic immune response induced by food proteins is characterized by the production of T-helper type 2 (Th2) cytokines, presence of antigen-specific serum IgE antibodies, and also an infiltration of eosinophils and an increase in the number of mast cells in the intestinal tract [1, 2]. Secreted antigen-specific IgE antibodies that are systemically distributed can bind to the high affinity receptor Fc*ε*RI on circulating basophils and tissue mast cells. After reexposure to the allergen, the cross-linking of IgE antibodies results in mast cell degranulation with the release of mediators such as histamine, cytokines, proteases, and platelet-activating factor (PAF), leading to both local and systemic symptoms [3].

PAF is a glycerophospholipid synthesized and secreted by mast cells, monocytes, and tissue macrophages. The binding of PAF to its receptor (PAFR) on platelets, monocytes, macrophages, and neutrophils results in many of the manifestations of anaphylaxis [4]. Anaphylaxis is considered an acute onset of food allergy marked by severe manifestations of immediate hypersensitivity reaction and it is known that PAF is an important mediator of this phenomenon. Although some studies show the effects of PAF specifically in anaphylaxis [4–6], there is a lack of studies about the role of PAF during the development of food allergy and its effects in the chronic consequences of this inflammatory process. We hypothesized that PAF might be also involved in other manifestations of food allergy.

In order to understand the role of PAF in the development and consequences of food allergy, we used a murine model of food allergy to ovalbumin (OVA) developed by our group, in which sensitized mice are orally challenged for 7 days with an OVA-containing diet (OVA diet). In this model, many signs similar to those presented by patients with food allergy are developed, such as antigenic aversion, increased anti-OVA IgE production, and intestinal eosinophil infiltration, as well as a marked weight and adipose tissue loss [7]. Moreover, we have demonstrated that this allergic process to OVA induces an adipose tissue inflammation with systemic metabolic consequences [8, 9]. Some studies have shown that there is a direct regulation by PAF in the inflammatory process that happens in adipose tissue, for example, during experimental obesity [10–12]. Therefore, the aim of this work was to elucidate both the role of PAF during the course of food allergy and the importance of this mediator in adipose tissue inflammation along with its alterations, as the change in the circulating levels of adipokines caused by OVA oral challenge after OVA-sensitization. To achieve our goal, we used mice lacking the PAF receptor and submitted them to an experimental model of food allergy.

## 2. Materials and Methods

### 2.1. Animals

Male BALB/c, six- to eight-week-old mice were obtained from the animal facility of Federal University of Minas Gerais (UFMG). PAF receptor deficient mice (PAFR^−/−^), with the same age as the wild type (WT) animals, were kindly provided by Professor Dr. Mauro Martins Teixeira, from the Immunology and Biochemistry department of UFMG. Mice received standard mouse chow diet (Purina) until the beginning of antigen challenge with OVA diet. The experiments were made according to the Ethical Principles in Animal Experimentation of our institution and the experimental protocol was approved by the Ethics Committee in Animal Experimentation of the University (protocol 85/2011-CETEA/UFMG).

### 2.2. OVA-Sensitization and Oral Challenge

OVA-sensitized mice (OVA+ group) received subcutaneous (sc) injection of 0.2 mL saline with 1 mg Al(OH)_3_, as adjuvant, and 10 *μ*g OVA (five times crystallized hen's egg albumin; Sigma, USA) on day 0 and saline with 10 *μ*g OVA on day 14. Mice from Control group (OVA−) received a sc injection of 0.2 mL saline with adjuvant on day 0 and saline on day 14. From day 21 until the end of the experiment the chow diet of all groups was replaced with a diet containing 14% of OVA. This diet was prepared using a lyophilized egg white (Salto's, Belo Horizonte, MG, Brazil) and the nutrient content was in accordance with AIN-93G [13].

### 2.3. Animal Evaluations and Tissue Collection

Body weight was determined daily during the OVA challenge. During the oral challenge period, OVA diet consumption was assessed daily by weighting the remaining chow and comparing its weight with the previous day. Data were reported as amount of diet consumption per group during the challenge week and divided by the number of animals per cage. Before the challenge starts and after 7 days of continuous OVA oral challenge, mice were anesthetized by intraperitoneal (ip) injection of 10 mg/kg xylazine and 100 mg/kg ketamine hydrochloride and blood was obtained from brachial plexus. Later, under deep anesthesia, mice were euthanized in order to collect the small intestine and perigonadal adipose tissue.

### 2.4. Disease Activity Index (DAI Score)

In order to assess the DAI score [8] the body weight loss and stool were evaluated every day during the OVA oral challenge according to the following scale. Body weight score: zero: no weight loss; one: 1–5% of weight loss; two: 6–10% of weight loss; three: 11–15% of weight loss; and four: >15% of weight loss; stool viscosity: zero: normal; two: fluffy; four: diarrhea.

### 2.5. Evaluation of Serum Anti-OVA IgE

Anti-OVA IgE ELISA was performed using plates coated with rat anti-mouse IgE (Southern Biotechnology Associates, Birmingham, USA), serum, and biotinylated OVA, as previously described [14]. The reaction was developed with the streptavidin-peroxidase conjugate (ExtrAvidin; Sigma), plus o-phenylene-diamine (OPD) and H_2_O_2_. The plate was read at 492 nm and the results were reported in arbitrary units (AU) using a positive reference serum as being 1000 units.

### 2.6. Histological Evaluation of Small Intestine

The proximal portion of the jejunum was fixed in 4% phosphate-buffered formaldehyde for 24 h. After fixation, the tissue was dehydrated in ethanol, cleared in xylene, and embedded in paraffin. Sections were cut (5 *μ*m) and stained with PAS technique (Periodic Acid-Schiff). Thus, goblet cells were evident in a dark pink tone. Three fields of images were captured per portion of the small intestine using a 10x objective of a microscope (Olympus Optical Co., Japan) equipped with a digital camera (Moticam 2500, China). These images were analyzed using the ImageJ software (National Institutes of Health, Maryland, USA), where all the green pixels were selected for the creation of a binarized image and subsequent calculation of the total area of goblet cells. The result was expressed in *μ*m^2^ PAS/field.

### 2.7. EPO Activity Assessment

After euthanasia, approximately 2 cm of the proximal jejunum was collected and frozen for posterior analyses. To measure eosinophilic peroxidase, 50 mg of samples were homogenized in 950 *μ*L of PBS and centrifuged at 4°C 12.000 ×g for 10 min. The supernatant was discarded and the erythrocytes were lysed. Samples were centrifuged again and the pellet was suspended in 950 *μ*L of 0.5% hexadecyltrimethyl ammonium bromide in PBS (HTAB solution). Samples were frozen and thawed three times in liquid nitrogen and centrifuged. The supernatant was diluted 3 times in PBS and used for the enzymatic assay. Briefly, o-phenylenediamine (OPD; 10 mg) was dissolved in 5.5 mL of distilled water and then 1.5 mL of OPD solution was added to 8.5 mL of Tris buffer (pH 8.0), followed by the addition of 7.5 *μ*L of H_2_O_2_. Using a 96-well plate (Microtest plates, Sarstedt, Germany), 100 *μ*L of substrate solution was added to 50 *μ*L of each sample. After 15 min, the reaction was stopped with 50 *μ*L of 1 M H_2_SO_4_ solution and the absorbance was read at 492 nm.

### 2.8. Histological Analysis of Perigonadal Adipose Tissue

Samples from perigonadal adipose tissue were fixed in 4% phosphate-buffered formaldehyde for 24 h, dehydrated in absolute ethanol, cleared in xylene, and then embedded in paraffin. Histological sections (5 *μ*m) were stained with hematoxylin-eosin (HE) and then evaluated by light microscopy (Olympus BX41). Images of four fields from each animal were captured using a digital camera coupled to a microscope (Olympus BX41) and the area of 50 adipocytes/mouse was measured using the ImageJ software (National Institutes of Health, Maryland, USA).

### 2.9. Intravital Microscopy of the Perigonadal Adipose Tissue Microvasculature

Mice were anesthetized with xylazine (10 mg/kg) and ketamine hydrochloride (100 mg/kg). The right jugular vein was cannulated and rhodamine 6G (Sigma, St. Louis, MO, USA) was injected intravenously (iv; 1.5 mg/kg) to label the leukocytes and endothelial cells. Then the perigonadal adipose tissue was collected by an abdominal incision. Rhodamine-epi-illumination was achieved with a 150 W variable HBO mercury lamp in conjunction with a Zeiss filter set 15 (546/12 nm band-pass filter, 580 nm Fourier transforms, 590 nm late potentials; Zeiss, Wetzlar, Germany). The microscopic images were captured using a Nikon eclipse 50i (Nikon Instruments Inc., Japan) microscope (×20 objective) with a video camera (5100 HS; Panasonic, Secaucus, NJ) and recorded digitally using both filter blocks consecutively. Data analysis was performed offline. Rolling leukocytes were considered as cells passing through a given point in the venule per minute. Adherent leukocytes were the cells that remained stationary for at least 30 s or longer within a 100 *μ*m length of venule. The two parameters analyzed were measured in three different vessels and the mean was obtained for each animal.

### 2.10. Determination of Serum Adipokines

Levels of serum adiponectin, resistin, and leptin were determined with DuoSet ELISA kits and they were performed according to the instructions provided by the manufacturer (R&D System, Inc., Minneapolis, USA).

### 2.11. Culture Assay

Before oral challenge, mice were euthanized and spleens were collected. Spleen cells were harvested in a RPMI 1640 medium (Sigma, USA) containing 10% fetal bovine serum and supplemented with 2 mM of L-glutamine, 10,000 of U penicillin, and 10 mg of streptomycin (Gibco, USA). Cells at 1 × 10^6^/well were then incubated in 24-well plates and treated with OVA (five times crystallized hen's egg albumin; Sigma, USA) (1000 *μ*g/mL) for 72 hours. Culture supernatants were tested for IL-4, IL-5, and IL-10 by ELISA using DuoSet ELISA kits according to the instructions provided by the manufacturer (R&D Systems, Inc., Minneapolis, USA).

### 2.12. Statistical Analysis

Results were expressed as mean ± standard error of the mean (SEM) for six mice in each group (each experiment was repeated twice) and analyzed using GraphPad Prism version 4.0 (GraphPad Software, San Diego, CA). The variance homogeneity of the data was tested with Bartlett's test. All data were analyzed for normality of distribution using the Kolmogorov-Smirnov test and were found to be normal. Parametric data were evaluated using one-way analysis of variance (ANOVA), followed by Newman-Keuls posttest. Differences were considered statistically significant at *p* < 0.05.

## 3. Results

### 3.1. Sensitized Mice Lacking the PAFR Showed Lower Specific Serum IgE after Oral Challenge with Consequent Decrease in Both Clinical Parameters and Intestinal Alterations Induced by Food Allergy

**Figure 1:**
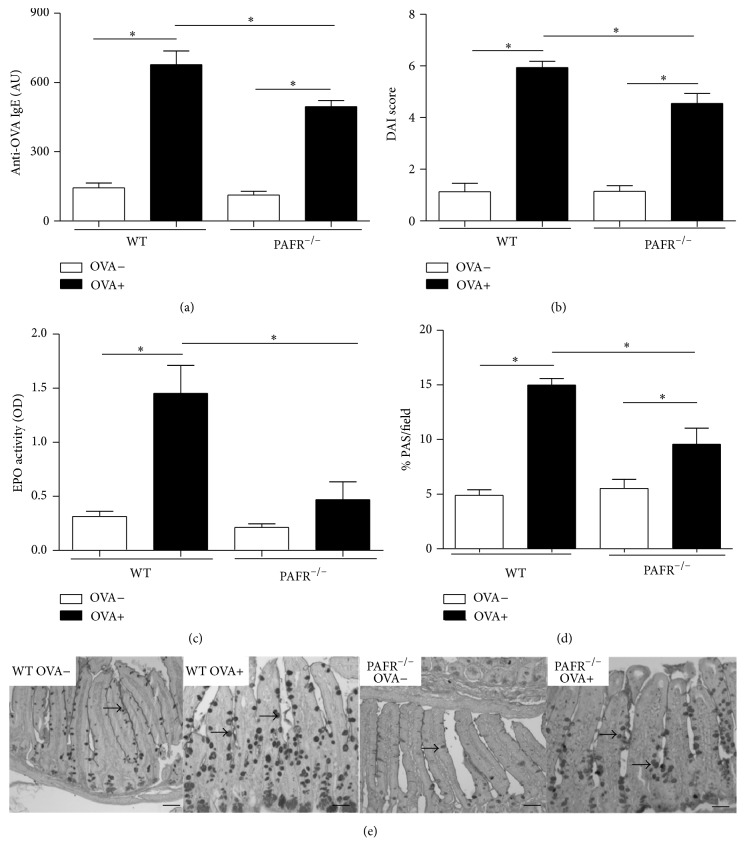
Markers of food allergy after 7 days of ovalbumin consumption by nonsensitized (OVA−) and sensitized (OVA+) mice. Serum anti-OVA IgE (a), DAI score (b), EPO activity in jejunum (c), and percentage of PAS (Periodic Acid-Schiff) by field also in jejunum histology (d). Representative photomicrographs of PAS stained (100x) intestine showing mucus production by goblet cells in evidence. Bars indicate 50 *μ*m. Data are expressed as mean ± SEM. ^*∗*^ *p* < 0.05.

As predicted by our experimental model of food allergy, sensitized mice after receiving OVA diet during one week (OVA+) showed higher serum anti-OVA IgE levels in comparison to nonsensitized animals that received the same diet (OVA−). Interestingly, levels of specific IgE in mice lacking PAFR from OVA+ group were significantly decreased in comparison to OVA+ WT mice (Figure 1(a)). These lower levels of anti-OVA IgE in PAFR-deficient mice were reflected in the DAI score, which represents the clinical parameters of the disease. These mice showed reduced clinical score in comparison to WT mice, both from OVA+ group. (Figure 1(b)).

Besides positive specific IgE titers, sensitized mice after receiving OVA diet were characterized by eosinophil infiltration and increased mucus production in the small intestine. When these features were analyzed in sensitized PAFR^−/−^ mice both parameters were significantly decreased in comparison to the OVA+ WT mice. OVA+ PAFR-deficient mice presented lower EPO activity (Figure 1(c)) and showed less mucus production in the small intestine in comparison to OVA+ WT mice (Figures 1(d) and 1(e)).

### 3.2. Lack of PAFR Reduced Both Body Weight and Adipose Tissue Loss Caused by Food Allergy

**Figure 2:**
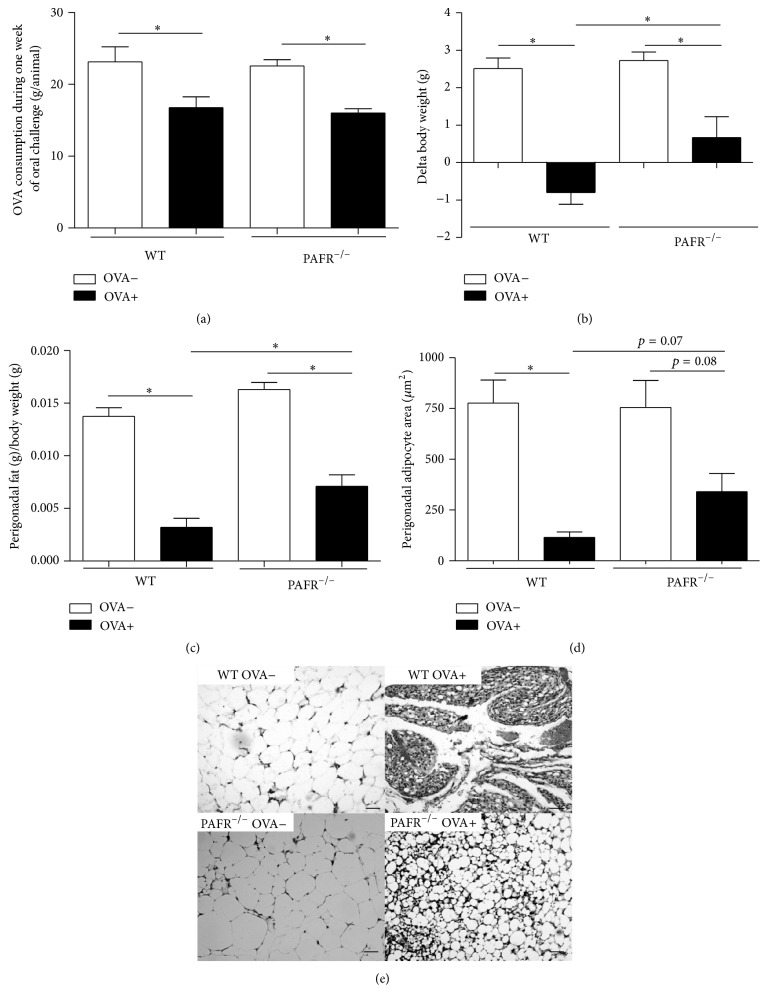
Ingestion of ovalbumin diet by sensitized (OVA+) and nonsensitized (OVA−) mice, mean of all consumption of the week challenge per animal (a). Variation of the body weight between the first and last day of oral challenge (b). Perigonadal adipose tissue weight (c) and adipocyte area from this tissue (d) after 7 days of OVA diet challenge. Representative photomicrographs of H&E stained (100x) perigonadal adipose tissue used to determine adipocyte areas (*μ*m^2^). Bars indicate 50 *μ*m. Data are expressed as mean ± SEM. ^*∗*^ *p* < 0.05.

One feature of our experimental model of food allergy is that sensitized mice followed by an oral challenge present a marked body weight and adipose tissue loss [8]. In fact, before the OVA challenge, there was no significant difference in body weight between sensitized (OVA+) and nonsensitized (OVA−) mice (data not shown), but 7 days after the oral challenge sensitized mice showed a remarkable loss in both body and adipose tissue weight (Figures 2(b) and 2(c)). Sensitized (OVA+) WT and PAFR-deficient mice consumed the same amount of OVA diet (Figure 2(a)), which was lesser than the amount consumed by nonsensitized mice (OVA−), because of the antigen aversion developed by allergic mice. Despite that, PAFR^−/−^ mice from the OVA+ group showed less body and adipose tissue loss compared to the OVA+ WT mice (Figures 2(b) and 2(c)). This adipose tissue loss observed in sensitized (OVA+) mice after oral challenge was followed by a decrease in adipocyte area from perigonadal adipose tissue and again this feature was not so marked in PAFR^−/−^ as WT mice (Figures 2(d) and 2(e)).

### 3.3. Despite Decreasing Classical Signs of Food Allergy, the Lack of PAFR Did Not Affect Resistin and Adiponectin Levels in Sensitized Mice after Oral Challenge

**Figure 3:**
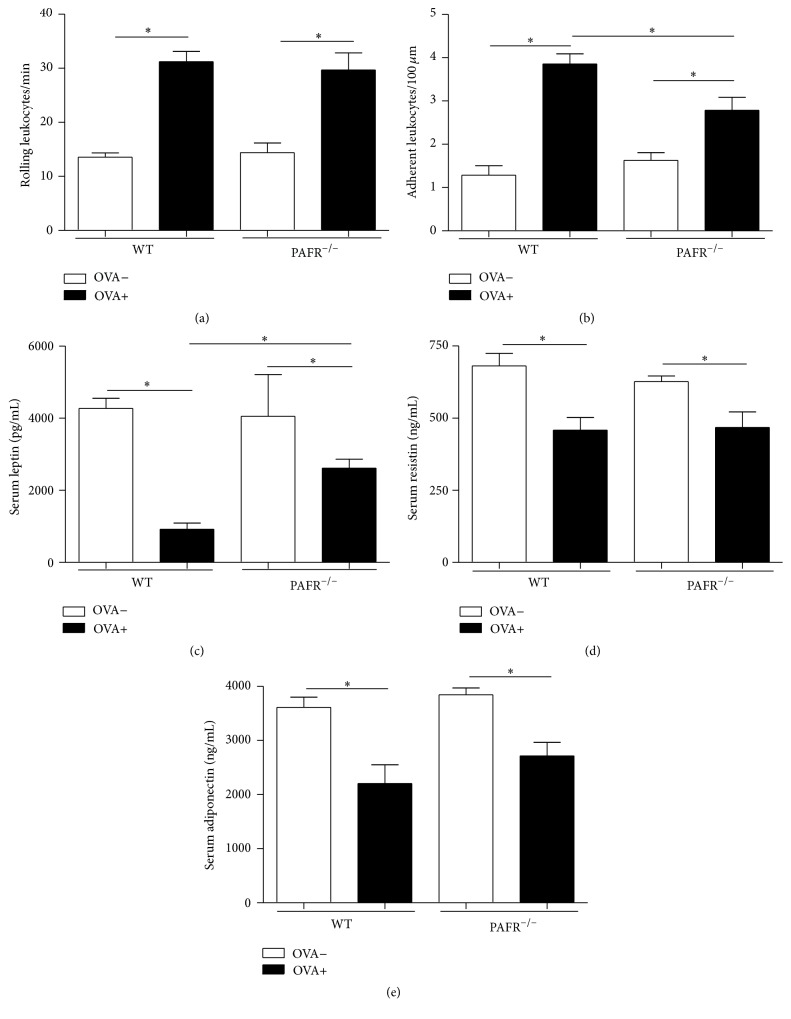
Visualization of leukocyte-endothelium interaction in the microvasculature of perigonadal adipose tissue in sensitized (OVA+) and nonsensitized (OVA−) mice after 7 days of oral OVA challenge. Intravital microscopy was used to assess the rolling (a) and adherent leukocytes (b). Levels of serum leptin (c), resistin (d), and adiponectin (e) were determined. Data are expressed as mean ± SEM. ^*∗*^ *p* < 0.05.

In order to assess the inflammation in the adipose tissue, intravital microscopy was performed. It was observed that sensitized (OVA+) mice, after 7 days of oral challenge, showed an increase in rolling and adherent leukocytes in the microvasculature of perigonadal adipose tissue in comparison to nonsensitized (OVA−) mice (Figures 3(a) and 3(b)). Sensitized (OVA+) mice lacking PAFR presented the same rolling leukocyte rate of OVA+ WT mice. However, the number of adherent leukocytes was significantly lower in these animals when compared to sensitized (OVA+) WT mice but it was still higher in comparison to nonsensitized (OVA−) mice.

In order to analyze the systemic effects of adipose tissue inflammation, levels of adipokines in the serum were determined. Adipokines are cytokines produced mainly by this tissue in response to different stimuli such as inflammation. Sensitized (OVA+) mice after 7 days of OVA challenge showed a significant decrease in the serum levels of serum leptin, resistin, and adiponectin (Figures 3(c), 3(d), and 3(e)). Interestingly, OVA+ PAFR^−/−^ mice after oral challenge also presented lower levels of serum resistin and adiponectin as WT OVA+ mice. However, serum levels of leptin in these animals, despite of being lower than nonsensitized (OVA−) mice, were significantly higher when compared to OVA+ WT mice.

### 3.4. Sensitized Mice Lacking PAFR Showed Lower Specific Serum IgE after Sensitization and Reduced Th2 Cytokine Response in OVA-Stimulated Splenocytes

**Figure 4:**
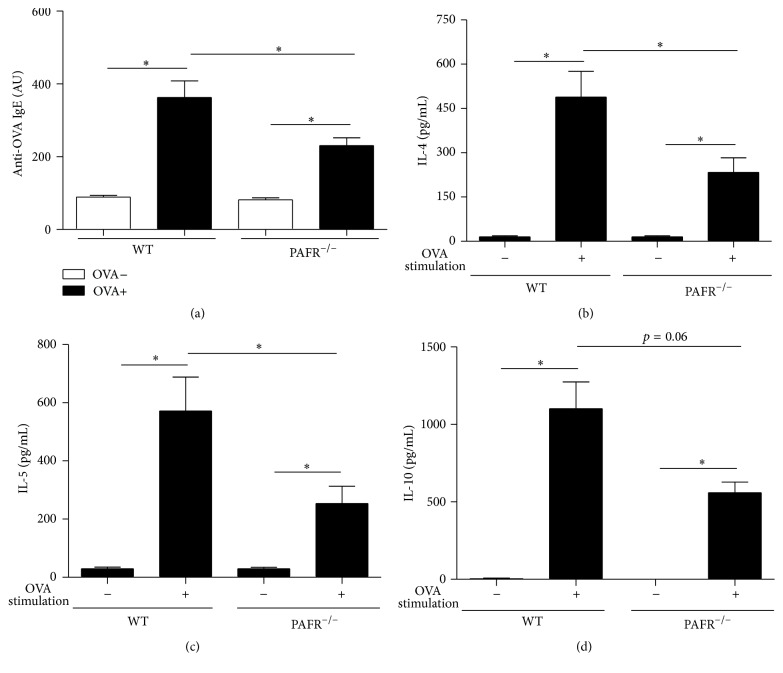
Serum anti-OVA IgE in nonsensitized (OVA−) and sensitized (OVA+) mice before oral ovalbumin challenge (a). Cytokine levels in supernatant of splenocyte culture from OVA-sensitized mice before oral challenge, IL-4 (b), IL-5 (c), and IL-10 (d). Data are expressed as mean ± SEM. ^*∗*^ *p* < 0.05.

In order to elucidate whether PAFR affects OVA-sensitization or operates during the effector phase of food allergy, the serum specific anti-OVA IgE was determined in sensitized animals before the oral challenge. As predicted by our experimental model of food allergy, sensitized mice (OVA+) showed higher serum anti-OVA IgE levels than nonsensitized animals (OVA−). Interestingly, levels of specific IgE of mice lacking OVA+ PAFR were significantly decreased in comparison to OVA+ WT mice (Figure 4(a)). To better understand how PAFR deficiency affects OVA-sensitization, spleen cells from sensitized animals were stimulated with OVA and the production of Th2 cytokines was analyzed. Cells from PAFR-deficient mice showed decreased production in IL-4 (Figure 4(b)), IL-5 (Figure 4(c)), and IL-10 (Figure 4(d)).

## 4. Discussion

Our data demonstrated that, after 7 days of oral challenge with OVA diet, OVA-sensitized mice lacking the PAF receptor showed decreased levels of serum anti-OVA IgE associated with attenuated allergic markers such as eosinophil infiltration and mucus production in the small intestine in comparison to OVA+ WT mice. Moreover, the body weight and adipose tissue loss followed by adipocyte area reduction was less expressive in PAFR-deficient mice. However, some features of inflamed adipose tissue presented by sensitized PAFR-deficient mice and WT mice after oral challenge were similar. OVA+ PAFR-deficient mice, as WT animals, showed a higher rate of rolling leukocytes in the microvasculature of adipose tissue, indicating cell activation. Also, circulating levels of adipokines such as resistin and adiponectin were decreased in comparison to nonsensitized mice and reached levels comparable to OVA+ WT mice.

IgE is an immunoglobulin responsible for type I hypersensitivity reactions and in our experimental model of food allergy we observe an increase in the levels of specific anti-OVA IgE in sensitized mice that is exacerbated after the oral challenge. In an interesting way, OVA+ PAFR-deficient mice showed significantly lower levels of circulating specific IgE when compared to OVA+ WT mice. This same profile of immunoglobulin was also observed after oral challenge by sensitized PAFR-deficient mice. Although it was already shown that the treatment with PAF antagonists is not able to change levels of IgE antibodies after OVA-sensitization [15], we observed a decrease in IgE levels. Probably that occurred because we used PAFR-deficient mice in which the absence of PAF signaling is complete, as oppesed to a partial depletion obtained with the antagonist treatment due to the dose used or even to the half-life of the drug. In addition, the experimental protocol in the mentioned study was different from ours, resulting in different levels of cell activation. Corroborating our data, a study demonstrated that PAF is important for proliferation and IgE secretion by human myeloma cells [16]. Also, it has been shown that IgE synthesis by human lymphocytes, evoked by IL-4, is enhanced by PAF, and PAF antagonists inhibit this potentiating effect [17]. Interestingly, spleen cells from sensitized PAFR-deficient mice after OVA-stimulation presented decreased Th2 response, such as production of IL-4, IL-5, and IL-10. These data indicate that the lower levels of specific IgE, after sensitization, could be a consequence of differential T cell activation since these cytokines are important for IgE production by B cells. These data are in accordance with the fact that PAF modulates an early event of T cell activation, enhancing T cell proliferation and leading to a substantial upregulation of IL-2 secretion and CD25 expression [18]. Moreover, there is a study showing that the expression of the PAFR is coupled to T cell activation and/or differentiation, highlighting its importance for the sensitization in our model [19]. Another hypothesis that could explain these low levels of specific IgE after oral challenge is the fact that once the allergic response was diminished in PAFR^−/−^ mice, the intestinal permeability caused by food allergy would be lower. Therefore, the lower intestinal permeability and the lower blood vessel dilatation caused by the absence of PAF culminates in a lower amount of OVA going through the intestinal mucosal barrier, causing less antigenic stimulation for IgE production.

Analyzing the clinical score of the disease (DAI score), OVA+ PAFR-deficient mice showed a lower score than OVA+ WT mice. This lower score was due to a not so intense weight loss from the PAFR-deficient mice, even though both groups showed the same level of antigen aversion, and to the diminished diarrheal response presented by these mice. Attenuated diarrheal response in the PAFR^−/−^ OVA+ group was expected because mast cells are required for experimental oral allergen-induced diarrhea due to the release and synergistic signaling induced by serotonin and PAF, but not histamine [20].

PAF is one of the most potent and versatile immune mediators found in mammals. Among its recognized functions are its ability to aggregate platelets and dilate blood vessels. Furthemore, among the nonregular functions exerted by PAF, we can mention its eosinophil chemotactic and activation capability [21, 22]. Supporting this data, sensitized PAFR-deficient mice, after oral challenge with OVA diet, showed significant decrease in the number and activity of eosinophils, measured by the quantification of eosinophilic peroxidase (EPO) in the jejunum compared with OVA+ WT mice after oral challenge. In addition, it has been demonstrated that specific granules from eosinophils in response to PAF release some cytokines such as eotaxin/CCL11 and IL-13 [23]. IL-13 is a key cytokine responsible for the induction of hyperplasia of goblet cells in allergic models [24]. Regarding mucus production, the same profile of EPO was presented, indicating that PAF is important for goblet cell activity and hyperplasia in this model in an indirect way.

Recent studies on PAF have shown that PAFR^−/−^ mice fed with either a high-fat diet or a high-refined carbohydrate-containing diet presented higher adiposity than wild type mice. These animals also presented a different inflammatory profile on adipose tissue along with its consequent metabolic alterations, demonstrating that PAF has an important role in these processes [10–12]. These results, together with the loss of adipose tissue followed by tissue inflammation in our model, led us to examine the effects of PAF deficiency in this context. We could observe that sensitized PAFR-deficient mice after oral challenge presented a lower adipose tissue loss, despite consuming a lower amount of diet comparable to WT sensitized mice because of the immunological aversion, showing that in these animals the adiposity was preserved. However, when we looked at the leukocyte recruitment into the microvasculature of adipose tissue, both PAFR-deficient and WT allergic mice showed the same rate of rolling leukocytes, showing that the level of activation, regarding the expression of molecules important for the rolling, was similar. Adipose tissue is a dedicated reservoir of functional mast cell progenitors and it is known that mast cell degranulation induces P-selectin-dependent leukocyte rolling and CD18-dependent leukocyte adhesion via histamine and PAF, respectively [25]. Therefore, because in allergic model mast cell degranulation is a common event and rolling leukocytes, as mentioned, are independent of histamine and not PAF, our result regarding rolling is in accordance with it. Moreover, another study showed that, during experimental autoimmune encephalomyelitis, PAF receptor is important in the induction of the disease but has no influence in the rolling steps of cell recruitment [26].

Furthermore, allergic PAFR^−/−^ mice levels of adipokines such as adiponectin and resistin, but not leptin, were reduced at the same levels of allergic WT mice. Leptin is an adipokine responsible for controlling the energetic homeostasis of the organism [27]. Moreover, there is a positive correlation between circulating levels of leptin and amount of adiposity. In fact, the lower reduction in adipose tissue weight on sensitized-OVA PAFR^−/−^ mice after oral challenge reflected in higher serum leptin levels in comparison to WT allergic mice. Regarding the circulating levels of resistin and adiponectin, they were previously described to be downregulated in our experimental food allergy model probably due to the high levels of TNF-*α* presented by adipose tissue from sensitized mice after oral challenge. Interestingly, allergic mice from WT and PAFR^−/−^ mice showed similar levels of these adipokines. This meant that PAF does not have a crucial role in the alteration of these adipokines in our model. In accordance with it, a study demonstrated that PAFR^−/−^ mice, after receiving a high-fat diet, despite showing a more severe adipose tissue inflammation, presented similar levels of adiponectin when compared to wild type mice that received the same diet, showing that this alteration is independent of the PAF signaling pathway [10]. In addition, a recent study shows that PAFR deficiency resulted in less inflammation in adipose tissue of mice fed a high-refined carbohydrate-containing diet, although the decrease in adiponectin level caused by this inflammation was unchanged in PAFR^−/−^ mice [11]. Moreover, adipose tissue is one depot rich in mast cells, especially during obesity and food allergy [8, 28]. Therefore, the specific IgE antibodies induced in OVA+ PAFR^−/−^ mice, even in lower levels than OVA+ WT mice, could result in an adipose tissue inflammation through mast cell activation that would be enough to maintain the decreased levels of adiponectin and resistin observed in our experimental model of food allergy.

## 5. Conclusions

In summary, our data showed that the absence of PAF signaling through PAFR results in a decrease of allergic response to OVA. However, PAF was not important to the altered profile of adipokines caused by oral ingestion of OVA after previous sensitization. Our work contributes towards clarifying some effects of PAF during food allergy development along with the role of this molecule in the metabolic consequences of this inflammatory process. We suggest that anti-PAF therapies could be an interesting way to treat food allergy but its consequences should be followed since some associated metabolic alterations remain unchanged with this approach.
